# Surface Characteristics and Fatigue Resistance of Ultrasonic Rolling-Treated 20Cr1Mo1V1A Valve Stem

**DOI:** 10.3390/mi16030265

**Published:** 2025-02-26

**Authors:** Shuailing Lan, Fan Chen, Wenbo Bie, Meng Qi, Zhiyuan Zhang

**Affiliations:** 1 School of Intelligent Manufacturing, Kaifeng University, Kaifeng 475003, China; lanshuailing@163.com; 2 Henan Province Engineering Research Center of Ultrasonic Technology Application, Pingdingshan University, Pingdingshan 467000, China; 15993149682@163.com; 3 School of Mechanical and Power Engineering, Henan Polytechnic University, Jiaozuo 454003, China; 4 School of Intelligent Manufacturing, Kaifeng Technician College, Kaifeng 454003, China

**Keywords:** ultrasonic rolling, 20Cr1Mo1V1A, surface characteristics, friction coefficient, corrosion

## Abstract

The valve stem made of 20CrMo1V1A has low surface resistance and high susceptibility to corrosion, significantly curtailing its service life. To address these issues, a high-quality ultrasonic rolling (USR) technology was applied to the 20CrMo1V1A stainless steel valve stem to enhance its corrosion resistance and mechanical properties. Subsequently, fatigue and corrosion tests were conducted on the valve stem. The results indicate that USR produces surfaces with a roughness average (Ra) of 0.3 μm and a gradient nanostructure on the valve stem surface. This unique microstructural modification resulted in a 27% improvement in surface hardness and nearly a three-fold grain size reduction. Additionally, the friction coefficient and electrochemical corrosion rate dropped by 47% and 32%, respectively. Therefore, USR was applicable for enhancing multiple properties of valve components as an additional final processing step for achieving high-performance valve stems.

## 1. Introduction

Valves are core components in aerospace, petrochemical, and natural gas applications [1,2]. The valve stem is crucial in determining the valve service life [3]. The material of 20CrMo1V1A is widely recognized as a predominant choice for manufacturing valve stems. However, its low surface properties, inadequate corrosion resistance, and unsatisfactory absolute strength continue to restrict its broader application [4,5]. Ultrasonic rolling (USR) is a surface strengthening technique that applies a gradient nanostructured layer to metal surfaces without altering their chemical composition [6]. In the research on the surface properties of URS, Zhang et al. [7] reported that pitting breakdown potential exhibited a significant negative shift due to USR treatment. Zhu [8] found that USR produced a thin layer with a refined microstructure that enhances surface quality. Bozdana [9] reported that USR improved fatigue life and resistance to failure mechanisms while maintaining geometric integrity. Cheng [10] assumed that USR processes further augment grain refinement and enhancements in surface hardness. Yang [11] studied how USR significantly contributes to material stability, increasing fatigue life by seven times. Zhao [12] investigated the impact of static force on surface integrity during USR and indicated that superior surface quality could be achieved within certain ranges of static force. Yao [13] established a linear correlation between rolling depth and rolling force during rotary ultrasonic burnishing. Ye [14] showed that USR can remarkably reduce surface roughness, refine grain structure, and substantially improve surface hardness. In the aspect of fatigue resistance properties of URS, Jia [15] indicates that URS significantly enhances the fatigue limit and life of welded joints, the fatigue strength of the welded components is increased by 2.04–4.58%, and a factor of 1.72–2.88 improves the corrosion fatigue life. Zou [16] indicates that the fatigue life of 7075 aluminum alloy is nearly tripled after ultrasonic rolling. Therefore, applying USR to the 20Cr1Mo1V1A valve stem to enhance valve performance is feasible. Initially, a hardened layer and a gradient nanostructure were prepared for the 20Cr1Mo1V1A valve stem. Subsequently, an investigation was conducted into the relationship between the surface microstructure and fatigue resistance of the valve stem, aiming to uncover the underlying mechanisms contributing to various performance enhancements.

## 2. Experimental

**Figure 1:**
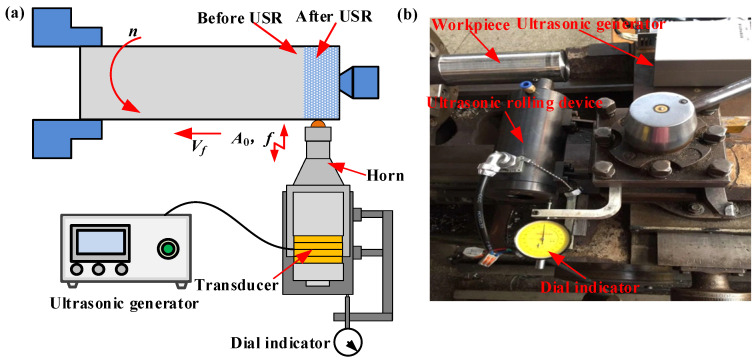
Experimental schematic illustration (**a**) and apparatus of the USR (**b**).

A series of samples exhibiting a hardness of 541 HV and a surface roughness of 4.3 μm were fabricated from the widely utilized high-performance valve stem material of 20Cr1Mo1V1A. The shafts had a diameter of 60 mm and a length of 250 mm. The machining schematic and experimental apparatus are illustrated in Figure 1. The workpiece rotates with the spindle, and different rolling depths are controlled by the lathe tool post. Along the axis of the workpiece, processing is carried out at intervals of 3 mm with different process parameters. The length of the processed workpiece is 10 mm, which serves as the specimen for the experiment.

The USR system was developed on the platform of a CA6120 lathe (Shenyang Yiji General Lathe Co., Ltd., Shenyang, China). The experimental setup comprised a 300-watt digital ultrasonic generator, a dial gauge, a 28 kHz transducer, and a conical horn with an output amplitude of 3 μm.

The relevant literature [17,18,19,20] indicate that rolling depth is a critical parameter influencing the surface integrity of materials. Consequently, only the rolling depth was considered during the research process; the primary USR parameters are summarized in Table 1.

Residual stress measurements were conducted directly using a PROTO-LXRD X-ray diffractometer (PROTO Manufacturing Ltd., Windsor, ON, Canada). An HV-1000 Vickers hardness tester (MH-5) (Ningbo Eeconomic and Technological Development zone Kaino Instrument Co., Ltd., Ningbo, China) was employed to assess surface hardness. The depth of the hardened layer and grain structure were detected using metallographic microscopy techniques. Finally, a Taylor Hobson roughness meter (Subtonic 3+) (Taylor-hobson, Leicester, UK) evaluated surface roughness and geometric morphology.

Friction and wear experiments were performed using model M-200A equipment produced by Beijing Zhongke Micro Nano Precision Instrument to evaluate frictional performance characteristics. A GCr15 steel ball with a diameter of 6.5 mm and hardness rating of 65 HRC was selected for these tests under conditions comprising an applied load of 0.5 N, frequency set at 10 Hz, over an experimental duration lasting one hour. Cut the workpiece into standard dimensions of 15 mm × 15 mm × 10 mm for testing. The surface to be tested should be polished to remove any surface contaminants and ensure uniformity. Place the ultrasonically rolled samples in a 3.5% NaCl solution for a specified period of 24 h.

## 3. Surface Characteristics

### 3.1. Residual Stress

**Figure 2:**
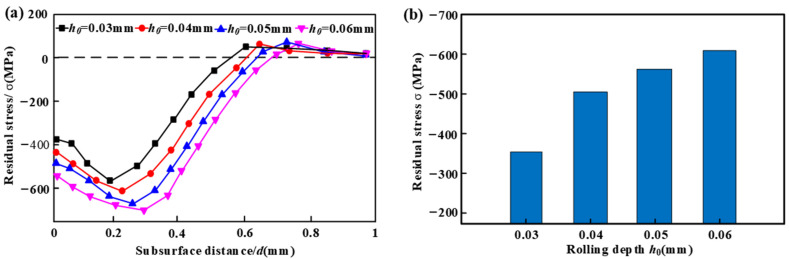
The effect of rolling depth on residual stress for the (**a**) subsurface and (**b**) surface.

The effect of rolling depth on residual stress is shown in Figure 2. Figure 2a illustrates residual stress distribution along the depth direction. The residual compressive stresses initially increase and subsequently decrease with increasing rolling depth. The maximum residual compressive stress occurs at the subsurface. For rolling depths of 0.03, 0.04, 0.05, and 0.06 mm, the peak locations of compressive residual stress are found at depths of 0.18, 0.23, 0.26, and 0.30 mm, respectively.

Figure 2b shows that surface residual compressive stress increases with greater rolling depth. This can be attributed to two factors: firstly, as the rolling depth increases, the energy from plastic strain transmitted to the workpiece by the roller also rises; this facilitates grain rearrangement and results in increased formation of residual compressive stress [21]. Secondly, an increase in rolling depth leads to a larger contact radius, which enhances shock frequency; this frequency is positively correlated with residual compressive stress [22]. Consequently, increasing rolling depth effectively enhances residual compressive stress levels. However, achieving significant residual compressive stress with minimal impact cycles is preferable from an efficiency standpoint.

### 3.2. Surface Roughness and Morphology

**Figure 3:**
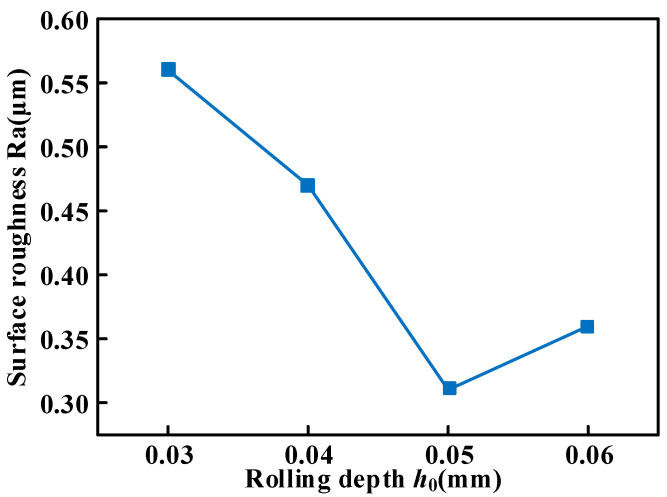
The effect of rolling depth on the surface roughness.

It can be observed from Figure 3 that as the rolling depth increases from 0.03 mm to 0.05 mm, the surface roughness by USR treatment decreases from 0.57 to 0.31 μm. However, when the rolling depth exceeds 0.05 mm, the surface roughness increases sharply to about 0.4 μm. It lies in the fact that surface roughness is greatly affected by the interaction between feed speed and contact radius. The increase improves the surface quality in the overlapping surface of the indentation caused by the rolling depth, which enhances the effect of peak shaving and valley filling. When the rolling depth exceeds 0.05 mm, the friction and wear between the workpiece surface and the roller will be intensified, which leads to unstable contact.

Figure 3 demonstrates that as the rolling depth increases from 0.03 to 0.05 mm, surface roughness resulting from USR treatment decreases from 0.57 to 0.31 μm. However, when exceeding a rolling depth of 0.05 mm, surface roughness sharply escalates to approximately 0.4 μm due to intensified interaction between feed speed and contact radius, affecting surface quality significantly. The improvement in surface quality arises from increased overlapping surfaces during indentation caused by deeper rolls, which enhance peak shaving and valley-filling effects. When surpassing a rolling depth of 0.05 mm, frictional wear between the workpiece’s surface and roller intensifies, leading to unstable contact conditions. Furthermore, the metal surface is significantly affected due to excessively long processing times, which results in a whorl shape on the surface and subsequently deteriorates its roughness.

**Figure 4:**
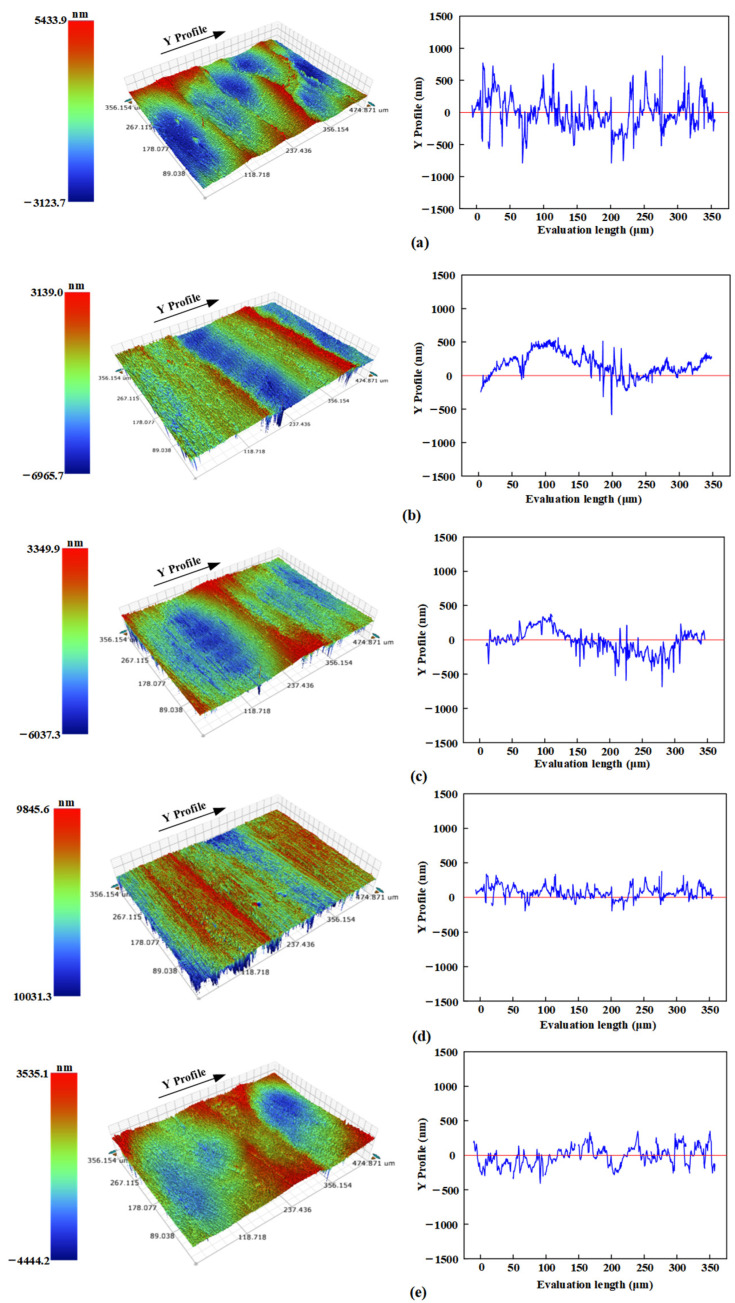
The effect of rolling depth on the morphology (**a**) *h*_0_ = 0 mm, (**b**) *h*_0_ = 0.03 mm, (**c**) *h*_0_ = 0.04 mm, (**d**) *h*_0_ = 0.05 mm and (**e**) *h*_0_ = 0.06 mm.

The impact of rolling depth on surface morphology is illustrated in Figure 4. As shown in Figure 4a–d, an increase in rolling depth leads to the gradual flattening of the surface topography. This phenomenon occurs because the workpiece surface experiences repeated impacts, enhancing the superposition effect of material peaks and valleys. The ripple width resulting from USR treatment has decreased from an initial value of 127 to 51 μm. The surface morphology observed at excessive rolling depths is depicted in Figure 4e. Large indentation areas cannot be completely overlapped when the rolling depth exceeds optimal levels, leading to a pronounced pit effect.

### 3.3. Hardness and Plastic Deformation Layer

**Figure 5:**
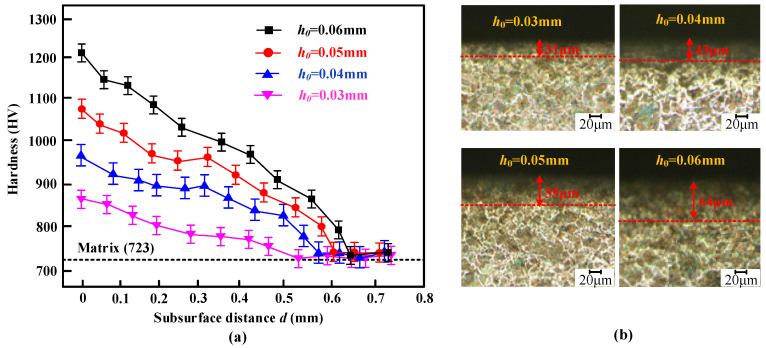
Hardness variation (**a**) and the plastic deformation layer (**b**).

Figure 5a presents the variation trend of hardness along the depth direction for 20Cr1Mo1V1A at different rolling depths. Compared to the matrix hardness, USR treatment effectively enhances the surface hardness of valve stems, which is consistent with that of [19]. With increasing rolling depth, sufficient plastic deformation occurs on the valve stem’s surface, resulting in a continuous rise in surface hardness and hardened layer thickness. At a rolling depth of 0.06 mm, the surface hardness reaches approximately twice that of the matrix (723 HV). Concurrently, there is a significant improvement in hardened layer depth; particularly at a rolling depth of 0.06 mm, this layer approaches a thickness of 0.73 mm 39%, greater than that achieved with a rolling depth of 0.03 mm.

Figure 5b illustrates the trend of the plastic deformation layer as a function of rolling depth. As the rolling depth increases, the thickness of the plastic deformation layer also increases. This phenomenon occurs because the contact area between the rolling head and the valve stem surface is relatively small, resulting in a rolling force that significantly exceeds the material’s yield limit. This excess force induces plastic deformation on the valve stem’s surface, contributing to a strengthening effect. Specifically, at rolling depths of 0.03, 0.04, 0.05, and 0.06 mm, the corresponding depths of the plastic deformation layer on the valve stem surface are 31, 43, 59, and 64 μm, respectively. The transition between the plastic deformation layer and the underlying matrix is smooth, exhibiting a symmetrical stepped distribution in the microstructure.

Additionally, under ultrasonic action, the microstructure at the top of the plastic deformation layer becomes denser, forming a distinct dense layer at a certain depth. This dense layer enhances the hardness of the surface layer, and the surface microstructure exhibits a flow direction aligned with the rolling feed direction. Notably, setting a rolling depth at approximately 0.05 mm constitutes approximately half of the total depth of the plastic deformation layer. Beyond this depth, the thickness of the dense layer reaches about 60% of the entire plastic deformation layer.

### 3.4. Gradient Grain Distribution

**Figure 6:**
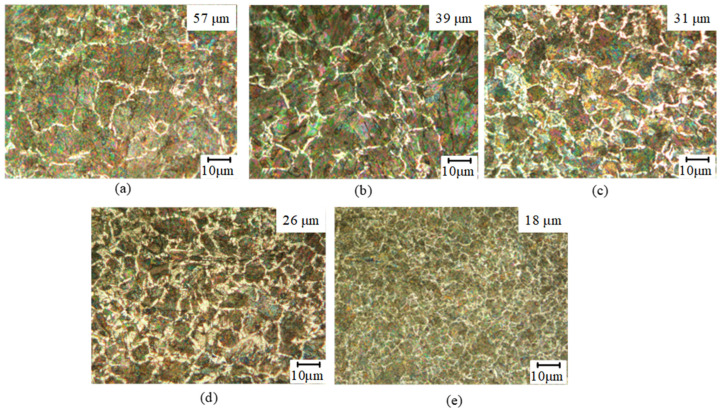
The effect of rolling depth on the grain (**a**) *h*_0_ = 0 mm, (**b**) *h*_0_ = 0.03 mm, (**c**) *h*_0_ = 0.04 mm, (**d**) *h*_0_ = 0.05 mm and (**e**) *h*_0_ = 0.06 mm.

It is evident from the Figure 6 that, in the absence of ultrasonic rolling treatment, the grain size exceeds 57 μm (Figure 6a), and there is no observable dislocation or slip phenomena. When applying rolling depths of 0.03 (Figure 6b), 0.04 (Figure 6c), 0.05 (Figure 6d), and 0.06 mm (Figure 6e), the grain sizes decrease to 39, 31, 26, and 18 μm, respectively. It is worth noting that when the rolling depth is 0.03 mm, the plastic strain energy begins to increase significantly, resulting in the expansion of the plastic flow area on the material’s surface per unit of time and the formation of obvious split lines in the grain. However, only a limited number of particles are segmented and refined. As the rolling depth continues to increase, there is a substantial rise in impacts per unit area, intensifying both plastic deformation and work hardening effects. Upon absorbing impact energy, larger grains begin to fracture near their boundaries while some grains become entangled and further refined. Once the rolling depth exceeds 0.05 mm, grain structures gradually approach a stable distribution pattern. The considerable infusion of ultrasonic vibration energy into these grains induces distortion and proliferation across numerous smaller grains on the valve stem’s surface; this process effectively reduces grain size and culminates in forming a stable transition layer characterized by uniformity and refinement.

## 4. Anti-Fatigue Characteristics

### 4.1. Friction Coefficient and Wear Amount

**Figure 7:**
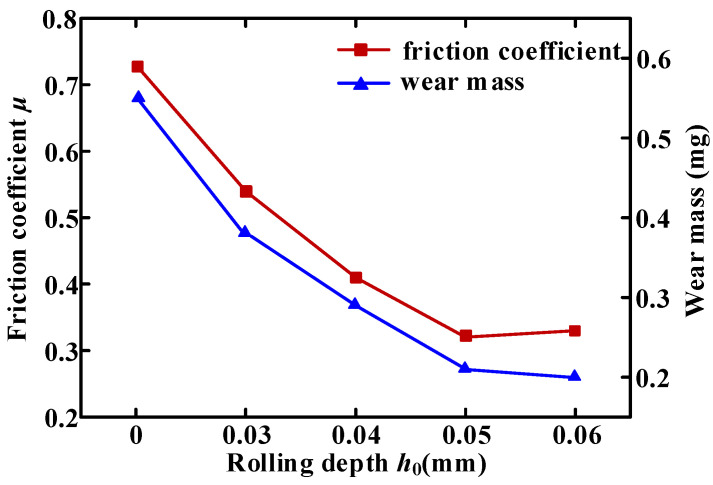
The effect of rolling depth on the friction coefficient and wear amount.

**Figure 8:**
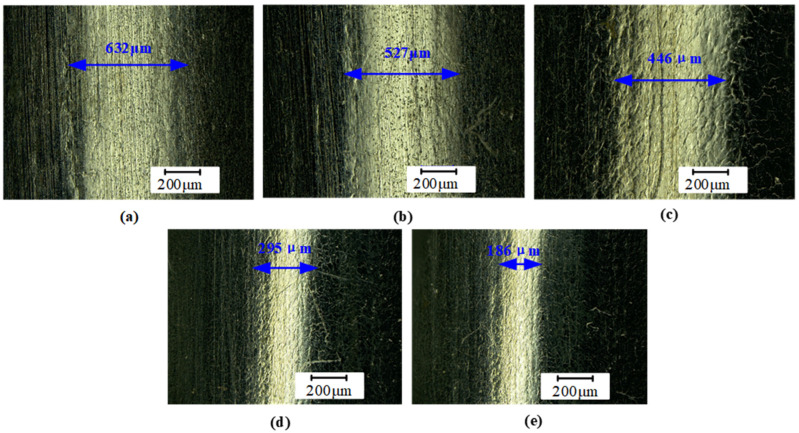
The effect of rolling depth on the friction and wear morphology (**a**) *h*_0_ = 0 mm, (**b**) *h*_0_ = 0.03 mm, (**c**) *h*_0_ = 0.04 mm, (**d**) *h*_0_ = 0.05 mm and (**e**) *h*_0_ = 0.06 mm.

The effect of rolling depth on friction coefficient and wear amount was shown in Figure 7. With the increasing rolling depth, the friction coefficient and wear amount decrease at first and then tend to be stable. The friction coefficient of the untreated sample was 0.72, and the wear amount was 0.53 mg. At a rolling depth of 0.03 mm, the friction coefficient of the sample dropped to 0.53, and the wear amount was reduced to 0.32 mg, decreasing by 36% and 52%, respectively. At a 0.05 mm rolling depth, the friction coefficient was the lowest, and the wear resistance of the 20Cr1Mo1V1A valve stem was the best. With the increasing rolling depth, friction coefficient and wear amount tended to balance. According to Figure 8a, the width of the abrasion on the untreated sample measures 632 μm. The material at the edge of the abrasion displays noticeable protrusions and plowing grooves, accompanied by a small number of granular debris. This phenomenon can be attributed to poor surface quality and relatively low hardness, characteristic features of typical abrasive wear. Figure 8b–e illustrates samples treated with USR, revealing a significant reduction in wear width, measuring 527, 446, 295, and 186 μm, respectively. These results indicate that USR is effective in diminishing wear width. Although samples treated with USR still exhibit characteristics associated with abrasive wear, their wear surfaces are notably smoother; furthermore, there is a substantial decrease in wear width and edge cracks and a reduction in the number of fatigue spalling pits compared to untreated samples. On the one hand, USR induces severe plastic deformation on the surface, significantly increasing surface hardness compared to traditional rolling. This enhanced hardness reduces the material’s susceptibility to wear, leading to lower wear amounts and narrower wear widths. On the other hand, USR promotes the formation of finer grains and subgrains on the surface. These finer grains have higher dislocation densities and increased mechanical strength, which further improve wear resistance. Traditional rolling may not achieve the same level of grain refinement, resulting in relatively higher wear. Thirdly, USR introduces residual compressive stress into the surface layer, which helps to close micro-cracks and prevent crack propagation during wear. This compressive stress enhances the material’s resistance to plastic deformation and wear, reducing wear amount and width. Traditional rolling may not generate the same level of residual compressive stress, resulting in higher wear. Finally, USR effectively reduces surface roughness by smoothing out micro-asperities, creating a more uniform surface finish. This reduction in roughness minimizes the contact area between the specimen and the counterface during wear, leading to lower friction and wear rates. Traditional rolling may not achieve the same level of surface smoothness, resulting in higher wear amounts and wider wear widths.

### 4.2. Corrosion Behavior

**Figure 9:**
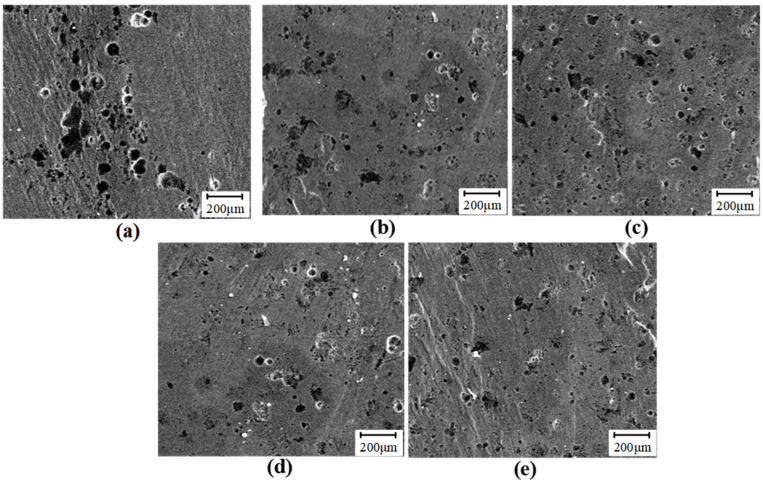
The effect of rolling depth on the corrosion morphology (**a**) *h*_0_ = 0 mm, (**b**) *h*_0_ = 0.03 mm, (**c**) *h*_0_ = 0.04 mm, (**d**) *h*_0_ = 0.05 mm and (**e**) *h*_0_ = 0.06 mm.

Figure 9 presents the surface corrosion morphology following a 48-h immersion in a 3.5 wt% NaCl solution across various rolling depths. Numerous corrosion pits are extensively distributed throughout the corroded surface, indicative of localized corrosion characteristics. In untreated samples, these corrosion pits are densely packed, almost covering the entire corroded area, resulting in more severe corrosive damage. Additionally, many diffuse aggregates of corrosion products can be observed on this surface. Conversely, corrosion pits for samples treated with USR appear relatively sparse; certain areas remain unaffected by the corrosive attack. Compared to untreated samples, the radius and area of these corrosion pits have been reduced by approximately half. Firstly, USR significantly reduces surface roughness by smoothing out micro-asperities and creating a more uniform surface finish. Lower surface roughness minimizes the formation of localized stress concentrations and micro-cracks, common initiation sites for corrosion pits. As a result, fewer corrosion pits form on the surface, and some areas remain unaffected by corrosion. Secondly, USR induces severe plastic deformation, leading to the formation of a nanostructured layer on the surface. This layer consists of fine grains and a high density of dislocations, enhancing the material’s corrosion resistance. Finally, USR introduces compressive residual stress into the surface layer. This compressive stress helps close micro-cracks and reduces the likelihood of pit initiation and propagation likelihood. The compressive stress also enhances the material’s resistance to plastic deformation, making it more difficult for corrosive ions to penetrate and initiate pitting corrosion. Traditional rolling may create localized stress concentrations and surface defects that act as initiation sites for corrosion pits. In contrast, USR produces a more uniform and refined surface, reducing these stress concentrations and minimizing the formation of corrosion pits. USR creates a gradient microstructure transitioning from nanostructured surface layers to the bulk material. This gradient structure enhances the overall mechanical and corrosion resistance properties by providing a more robust barrier against corrosive attack.

## 5. Conclusions

This study successfully applied USR to a 20Cr1Mo1V1A valve stem to enhance its surface anti-fatigue properties. The principal conclusions are as follows:

(1) When the rolling depth was precisely set at 0.05 mm, the surface roughness reached an optimal level of 0.32 μm. At this depth, several key surface and subsurface properties were significantly enhanced. The surface hardness was measured at 1087 Hv, while the residual compressive stress reached −489 MPa. Both the deep hardening layer and the plastic deformation layer extended to a depth of 59 μm. The grain size was also reduced to approximately 26 μm, with coarser grains observed at greater depths.
(2) Ultrasonic rolling significantly improved the wear resistance of the 20Cr1Mo1V1A valve stem. Compared to the untreated sample, the friction coefficient and wear mass were reduced by 57% and 69%, respectively. Moreover, the friction width was substantially decreased from 632 μm to 195 μm.
(3) The corrosion resistance of the 20Cr1Mo1V1A valve stem treated with ultrasonic rolling was notably superior to that of the untreated samples when exposed to a 3.5 wt% NaCl aqueous solution. Specifically, the radius and area of corrosion pits were approximately halved compared to those of the untreated samples.

## Figures and Tables

**Table 1:** USR processing parameters.

Rolling Depth				Feed Rate	Spindle	Roller Radius	Frequency	Amplitude
*h*_0_/mm				*v_f_*/mm·r^−1^	*n*/mm·r^−1^	R_T_/mm	*f*/kHz	*A*_0_/μm
0.03	0.04	0.05	0.06	0.1	100	3	28	3

## Data Availability

All data generated or analyzed during this study are included in the present article.
